# Regulatory Role of GRK2 in the TLR Signaling-Mediated iNOS Induction Pathway in Microglial Cells

**DOI:** 10.3389/fphar.2019.00059

**Published:** 2019-02-04

**Authors:** Sailesh Palikhe, Wakana Ohashi, Takuya Sakamoto, Kohshi Hattori, Masaaki Kawakami, Tsugunobu Andoh, Hiromi Yamazaki, Yuichi Hattori

**Affiliations:** ^1^Department of Molecular and Medical Pharmacology, Graduate School of Medicine and Pharmaceutical Sciences, University of Toyama, Toyama, Japan; ^2^Department of Anesthesiology and Pain Relief Center, The University of Tokyo Hospital, Tokyo, Japan; ^3^Department of Applied Pharmacology, Graduate School of Medicine and Pharmaceutical Sciences, University of Toyama, Toyama, Japan; ^4^Faculty of Nursing Science, Tsuruga Nursing University, Tsuruga, Japan; ^5^The Research Institute of Cancer Prevention, Health Sciences University of Hokkaido, Tobetsu, Japan

**Keywords:** GRK2, iNOS, TLR signaling, interferon-β, IRF1, STAT pathway, microglia

## Abstract

G protein-coupled receptor kinase 2 (GRK2) is a ubiquitous member of the GRK family that restrains cellular activation by G protein-coupled receptor (GPCR) phosphorylation leading to receptor desensitization and internalization, but has been identified to regulate a variety of signaling molecules, among which may be associated with inflammation. In this study, we attempted to establish the regulatory role of GRK2 in the Toll-like receptor (TLR) signaling pathway for inducible nitric oxide synthase (iNOS) expression in microglial cells. When mouse MG6 cells were stimulated with the TLR4 ligands lipopolysaccharide (LPS) and paclitaxel, we found that interferon regulatory factor 1 (IRF1) protein expression and activation was upregulated, transcription of interferon-β (IFN-β) was accelerated, induction/activation of STAT1 and activation of STAT3 were promoted, and subsequently iNOS expression was upregulated. The ablation of GRK2 by small interfering RNAs (siRNAs) not only eliminated TLR4-mediated upregulation of IRF1 protein expression and nuclear translocation but also suppressed the activation of the STAT pathway, resulting in negating the iNOS upregulation. The TLR3-mediated changes in IRF1 and STAT1/3, leading to iNOS induction, were also abrogated by siRNA knockdown of GRK2. Furthermore, transfection of GRK2 siRNA blocked the exogenous IFN-β supplementation-induced increases in phosphorylation of STAT1 as well as STAT3 and abrogated the augmentation of iNOS expression in the presence of exogenous IFN-β. Taken together, our results show that GRK2 regulates the activation of IRF1 as well as the activation of the STAT pathway, leading to upregulated transcription of iNOS in activated microglial cells. Modulation of the TLR signaling pathway via GRK2 in microglia may be a novel therapeutic target for treatment of neuroinflammatory disorders.

## Introduction

Lipopolysaccharide (LPS), a glycolipid constituent of the outer membrane of Gram-negative bacteria, initiates inflammatory signaling cascades in cells, including monocytes, macrophages, dendritic cells, and endothelial cells, leading to the upregulation of cytokines, chemokines, and other inflammatory mediators, such as inducible nitric oxide synthase (iNOS) and cyclooxygenase-2 (COX-2). As critical pattern recognition receptors for the first line of the host defense system against bacteria, viruses, fungi, and parasites, Toll-like receptors (TLRs) are widely found on the surface of those cells and play a key role in the innate immune system (Takeda and Akira, 2003; Beutler, 2009; Kawai and Akira, 2010; Satoh and Akira, 2016). Among these TLRs, TLR4 is activated by LPS, which is primarily associated with the accessory protein MD-2 and the co-receptor CD-14 to recognize LPS, resulting in transducing signals for activation of several transcription factors, such as nuclear factor-κB (NF-κB), in cooperation with myeloid differentiation factor 88 (MyD88) (Beutler, 2009). MyD88 leads to activation of the serine/threonine kinase interleukin-1 receptor-associated kinase 4 (IRAK4), which engages with mitogen-activated protein kinase (MAPK) cascades (extracellular signal-regulated protein kinase [ERK], c-Jun N-terminal kinase [JNK], and p38) and results in NF-κB activation and subsequent upregulation of expression of pro-inflammatory mediators (Byrd-Leifer et al., 2001; Guha and Mackman, 2001; Suzuki and Saito, 2006; Kawai and Akira, 2010; Satoh and Akira, 2016). Alternatively, the phosphoinositide 3-kinase (PI3K)/Akt pathway downstream of TLR4 signaling could also induce NF-κB activation (Li et al., 2003; Rosadini and Kagan, 2017). It should be also noted that the canonical NF-κB pathway responds to diverse stimuli, including TLR activation, is activated with the inducible degradation of IκBα (inhibitor of κBα) triggered through its site-specific phosphorylation by multi-subunit IκB kinase complex, and participates in the induction of type I interferons (IFNs) and pro-inflammatory cytokines (Shin et al., 2015; Liu et al., 2017).

G protein-coupled receptor kinases (GRKs) are serine/threonine kinases that were originally identified to phosphorylate activated G protein-coupled receptors (GPCRs) and cause desensitization of GPCR signaling (Premont and Gainetdinov, 2007; Ribas et al., 2007). The seven mammalian GRKs can be divided into three subfamilies based on sequence and functional similarities: the rhodopsin kinase or visual GRK subfamily (GRK1 and GRK7), the β-adrenergic receptor kinase subfamily (GRK2/GRK3), and the GRK4 subfamily (GRK4, GRK5, and GRK6) (Ribas et al., 2007). Among them, the ubiquitous isoform GRK2, also known as β-adrenergic receptor kinase-1 (βARK1), has been documented to regulate other pathways independently of its role in GPCR phosphorylation (Ribas et al., 2007; Jurado-Pueyo et al., 2008; Penela et al., 2014). Thus, GRK2 can restrain cellular signaling via direct interaction with downstream kinases such as Akt, MAPK kinases 1 and 2 (MEK1/2), PI3K, and p38 MAPK, leading to inhibition of their activities (Reiter and Lefkowitz, 2006; Ribas et al., 2007; Penela et al., 2010; Evron et al., 2012). Incoming evidence suggests a key role of GRK2 in the inflammatory signaling pathways. Intriguingly, GRK2 has been reported to be highly expressed in the immune system being a critical regulator of inflammatory responses (Vroon et al., 2006). Furthermore, mice with GRK2 depletion in cells of myeloid lineage appear to display exaggerated inflammatory cytokine/chemokine production and organ injury as a result of macrophage hyperreaction to endotoxemia (Patial et al., 2011). Besides, it is of interest to note that GRK2 levels are altered in immune cells from human patients with some inflammatory disorders (Lombardi et al., 1999, 2001; Giorelli et al., 2004; Vroon et al., 2005; Arraes et al., 2006; Cruces-Sande et al., 2018).

Our recent work has shown that GRK2 plays a critical role in iNOS gene transcription in microglial cells stimulated with LPS (Kawakami et al., 2018). Based on this result, we postulated that GRK2 may function as TLR signaling to induce iNOS expression. To test this hypothesis, we attempted to delineate the role and mechanisms by which GRK2 regulates the TLR signaling pathway for iNOS induction using cultured mouse MG6 microglial cells.

## Materials and Methods

### Cell Culture and Reagent

Mouse microglial cell line MG6 cells (RCB2403) were obtained from RIKEN BRC (Tsukuba, Japan) and cultured as described previously (Kawakami et al., 2018). Cells were maintained until 70% confluency in Dulbecco’s modified Eagle’s medium containing 10% fetal bovine serum, 10 μg/ml insulin, 10 μM 2-mercaptoethanol, 100 U/ml penicillin, and 100 μg/ml streptomycin at 37^°^C in a 5% CO_2_ incubator. Phosphorothioate-modified oligodeoxynucleotide (ODN) 1668 and control ODN were synthesized by Hokkaido system science (Hokkaido, Japan). The ODN1668 sequence was TCCATGACGTTCCTGATGCT and used as CpG-ODN. The control ODN (CTL-ODN) sequence was TCCATGAGCTTCCTGATGCT. GRK2 inhibitor (GRK2i), methyl 5-[2-(5-nitro-2-furyl)vinyl]-2-furoate was purchased from Calbiochem (San Diego, CA, United States). Stattic was obtained from Abcam (Cambridge, United Kingdom). BAY11-7082 and MG-132 were purchased from Sigma (Sigma, St. Louis, MO, United States).

### siRNA Transfection

All small interfering RNAs (siRNAs) were purchased from Sigma-Aldrich (St. Louis, MO, United States). MISSION siRNA universal negative control (SIC-001) was employed as the negative control in this study. GRK2 siRNAs, signal transducers and activators of transcription 1 (STAT1) siRNAs, IFN regulatory factor 1 (IRF1) siRNAs, TIR-domain-containing adaptor-inducing interferon-β (TRIF) siRNAs, STAT3 siRNAs, interferon alpha and beta receptor subunit 1 (IFNAR1) siRNAs and IRF3 siRNAs were transfected at a final concentration of 60, 15, 50, 50, 40, 40, and 50 nM using lipofectamine RNAiMAX (Life Technologies, Carlsbad, CA, United States) according to the manufacturer’s protocol, respectively.

### Western Blot Analysis

Cells were harvested and lysed in 300 μl of Radio-Immunoprecipitation Assay (RIPA) buffer (Thermo Fisher Scientific, Rockford, IL, United States) containing protease inhibitor cocktail (Nacalai Tesque, Kyoto, Japan) on ice. The lysates were centrifuged at 18,000 ×*g* for 10 min at 4°C and the resulting supernatants were reserved. The supernatant proteins were quantified using BCA Protein Assay Kit (Thermo Fisher Scientific). Samples (20 μg of protein) were run on 10% polyacrylamide gel and electrotransferred onto polyvinylidene fluoride filter membrane. The membrane was blocked for 60 min at room temperature in 1% bovine serum albumin in Tris-buffered saline containing Tween 20, followed by overnight incubation with primary antibody, anti-iNOS rabbit polyclonal antibody (1:1,000; Cell Signaling, Danvers, MA, United States), anti-STAT1 mouse monoclonal antibody (1:300; Santa Cruz Biotechnology, Santa Cruz, CA, United States), anti-phospho-STAT1 (Tyr-701) mouse monoclonal antibody (1:300; Santa Cruz Biotechnology), anti-GRK2 rabbit polyclonal antibody (1:500; Santa Cruz Biotechnology), anti-STAT3 mouse monoclonal antibody (1:1000; Cell Signaling), anti-phospho-STAT3 (Tyr-705) rabbit monoclonal antibody (1:1000; Cell Signaling), anti-IRF1 rabbit monoclonal antibody (1:1000; Cell Signaling), anti-lamin B1 rabbit polyclonal antibody (1:3000; Proteintech, Rosemont, IL, United States), or anti-glyceraldehyde-3-phosphate dehydrogenase (GAPDH) mouse monoclonal antibody (1:10000; Wako Pure Chemical, Osaka, Japan), at 4°C. Primary antibody detection was performed with horseradish peroxidase-conjugated secondary antibodies. Binding of the antibody was detected by an enhanced chemiluminescence (ECL) Plus chemiluminescent system (GE Healthcare, Tokyo, Japan) and levels of protein expression were quantified by a lumino image LAS-4000 analyzer (Fuji Film, Tokyo, Japan). Additional details are described by our laboratory (Sakata et al., 2015; Abdelzaher et al., 2016; Ohashi et al., 2017; Kawakami et al., 2018).

### RNA Extraction and Quantitative Reverse-Transcribed PCR

Total RNA was isolated from cells with the use of Sepazol-RNA I Super G (Nacalai Tesque) according to the manufacturer’s manual. ReverTra Ace qPCR RT Master Mix (Toyobo, Osaka, Japan) was used for the reverse transcription reaction, and quantitative PCR analyses were performed using PowerUp^TM^ SYBR^®^ Green Master Mix (Thermo Fisher Scientific), as described in the manufacturers’ instructions. Values were normalized to the housekeeping gene GAPDH according to the manufacturer’s protocol (MX3000P real-time PCR system; Agilent Technologies Inc., Santa Clara, CA, United States). The IFN-β primer sequences were 5′-CAGCTCCAAGAAAGGACGAAC-3′ (sense) and 5′- GGCAGTGTAACTCTTCTGCAT-3′ (antisense), the iNOS primer sequences were 5′-TAGGCAGAGATTGGAGGCCTTG-3′ (sense) and 5′-GGGTTGTTGCTGAACTTCCAGTC-3′ (antisense), the IRF1 primer sequences were 5′-ATGCCAATCACTCGAATGCG-3′ (sense) and 5′-TTGTATCGGCCTGTGTGAATG-3′ (antisense), the interferon-γ-inducible 10 kD protein (IP10) primer sequences were 5′-CCAAGTGCTGCCGTCATTTTC-3′ (sense) and 5′-GGCTCGCAGGGATGATTTCAA-3′ (antisense), the interleukin (IL) -6 primer sequences were 5′-CCACTTCACAAGTCGGAGGCTTA-3′ (sense) and 5′-GCAAGTGCATCATCGTTGTTCATAC-3′ (antisense), the IL-1β primer sequences were 5′-TCCAGGATGAGGACATGAGCAC-3′ (sense) and 5′-GAACGTCACACACCAGCAGGTTA-3′ (antisense), the IRF7 primer sequences were 5′-GAGACTGGCTATTGGGGGAG-3′ (sense) and 5′-GACCGAAATGCTTCCAGGG-3′ (antisense), and the GAPDH primer sequences were 5′-TGTGTCCGTCGTGGATCTGA-3′ (sense) and 5′-TTGCTGTTGAAGTCGCAGGAG-3′ (antisense). Additional details are described elsewhere (Kawakami et al., 2018; Yamashita et al., 2018).

### Enzyme Immunoassay for IFN-β

Culture medium levels of IFN-β were measured by the use of commercially available enzyme-linked immunosorbent assay (ELISA) kit (Mouse IFNβ DuoSet ELISA kit, DY8234-05; R&D Systems, Minneapolis, MN, United States) according to the manufacturer’s instructions. The plate was read on a microplate reader (Molecular Devices, Menlo Park, CA, United States). Assays were performed in duplicate.

### FACS Analysis

Microglial cells were stimulated with 100 ng/ml of LPS for 3 h. The cells were incubated with allophycocyanin (APC)-anti-TLR4 (Clone: SA15-21) for 15 min on ice and then stained with SYTOX-ADDvanced. Fluorescence-activated cell scanning (FACS) analysis by flow cytometry was performed on an Accuri C6 (Becton Dickinson, Franklin Lakes, NJ, United States). The FACS pattern obtained from SYTOX-ADD-negative cells is indicated.

### Statistics

Values are presented as mean ± SEM. Data were analyzed by the use of Prism software (version 6; GraphPad Software, San Diego, CA, United States). Statistical significance was calculated by Student’s unpaired *t*-test, where differences at *p* < 0.05 was considered statistically significant.

## Results

### STAT1/3 Activation Is Involved in iNOS Expression in Microglia

**FIGURE 1:**
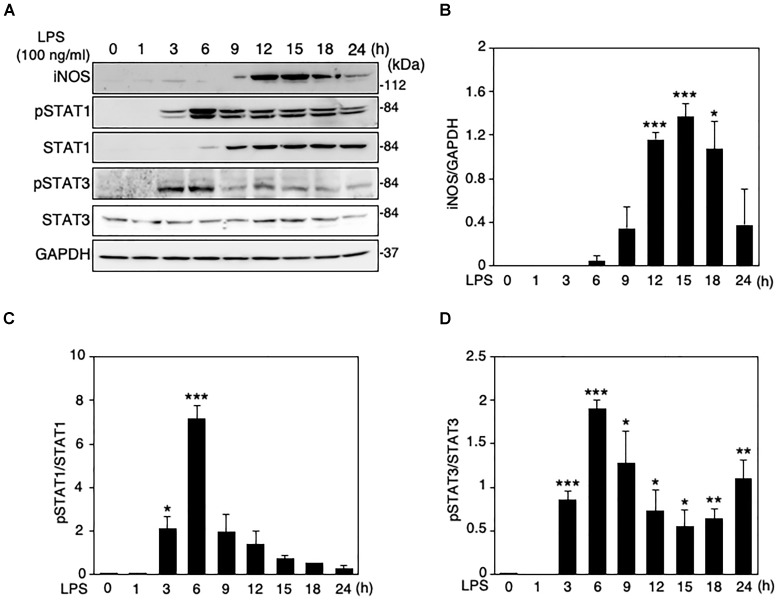
Changes in protein expression levels of iNOS and total and phosphorylation levels of STAT1 and STAT3 in LPS-stimulated MG6 cells. **(A)** Typical Western blots of iNOS, phospho-STAT1 at Tyr-701, total STAT-1, phospho-STAT3 at Tyr-705, and total STAT-3 after challenge with 100 ng/ml LPS. GAPDH served as loading control. **(B)** Time course of changes in iNOS protein expression after LPS application. **(C)** Time course of changes in STAT1 phosphorylation after LPS application. **(D)** Time course of changes in STAT3 phosphorylation after LPS application. The results represent the mean ± SEM for three independent experiments. ^∗^*P* < 0.05, ^∗∗^*P* < 0.01, and ^∗∗∗^*P* < 0.001 vs. time 0 by *t*-test.

When LPS (100 ng/ml) was applied to the mouse microglial cell line MG6 cells, the expression levels of iNOS protein were increased in a time-dependent manner, reaching a peak at 15 h after LPS and declining thereafter (Figure 1A,B). The induction of iNOS expression in glial cells and macrophages involves the activation of the Janus tyrosine kinase (JAK)/STAT signaling pathway (Dell’Albani et al., 2001; Ganster et al., 2001). We thus ascertained whether STAT1 and STAT3 can be activated in MG6 cells following LPS challenge. Activation of STAT1 and STAT3 was assessed by Western blot analysis for phospho-STAT1 at Tyr-701 and phospho-STAT3 at Tyr-705, respectively. STAT1 phosphorylation was transiently but markedly increased in cells at 6 h after LPS, whereas a sustained rise in total STAT1 levels was also observed with LPS stimulation (Figure 1A,C). On the other hand, the increase in STAT3 phosphorylation showed a marked peak at 6 h after LPS and a less pronounced sustained response, but total STAT3 levels were unaffected by LPS challenge (Figure 1A,D).

**FIGURE 2:**
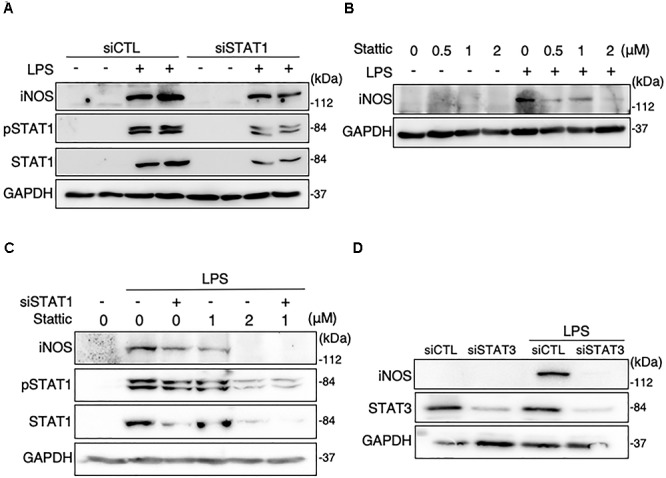
Effects of STAT1 siRNA transfection and stattic on protein expression levels of iNOS and total and phosphorylation levels of STAT1 in LPS-stimulated MG6 cells. **(A)** iNOS, phospho-STAT1 at Tyr-701, and total STAT1 before and 15 h after 100 ng/ml LPS application in the presence of or STAT1 siRNAs (siSTAT1) or the negative control siRNAs (siCTL). **(B)** Concentration-dependent effect of stattic (0.5–2 μM) on iNOS expression before and 15 h after 100 ng/ml LPS application. **(C)** Effects of siSTAT1 alone, stattic (2 μM) alone, and combination of siSTAT1 and stattic (1 μM) on iNOS, phosphorylated STAT1, and total STAT1 before and 15 h after 100 ng/ml LPS application. **(D)** Effect of STAT3 siRNAs (siSTAT3) on iNOS and STAT3 expression before and 15 h after 100 ng/ml LPS application. GAPDH served as loading control. Shown are representative Western blots from three independent experiments in which the same results were obtained.

The knockdown of STAT1 was conducted in MG6 cells using its specific siRNAs. Our transfection of STAT1 siRNAs effectively silenced STAT1 expression levels in LPS-stimulated microglial cells (Figure 2A). Transfection of STAT1 siRNAs evidently but incompletely prevented the LPS-induced increase in iNOS protein expression (Figure 2A). Stattic is known to be a small-molecule inhibitor of STAT3 activation, dimerization, and nuclear translocation (Schust et al., 2006). Treatment with stattic reduced iNOS protein expression in LPS-stimulated cells in a concentration-dependent manner (Figure 2B). At a concentration of 2 μM, stattic completely eliminated the LPS-induced iNOS upregulation, but also abolished the total and phosphorylation levels of STAT1 following LPS application (Figure 2B,C), suggesting that stattic at this concentration appears to act on STAT1 as well as STAT3. Combined treatment with STAT1 siRNAs and stattic at a lower concentration (1 μM) resulted in a complete abolition of iNOS expression in LPS-stimulated cells (Figure 2C). We also found that iNOS expression following LPS challenge was inhibited when STAT3 siRNAs were transfected (Figure 2D). These results indicate that both STAT1 and STAT3 play a role in iNOS expression triggered by LPS in microglial cells.

### GRK2 Regulates STAT1 and STAT3 Phosphorylation in Microglia

**FIGURE 3:**
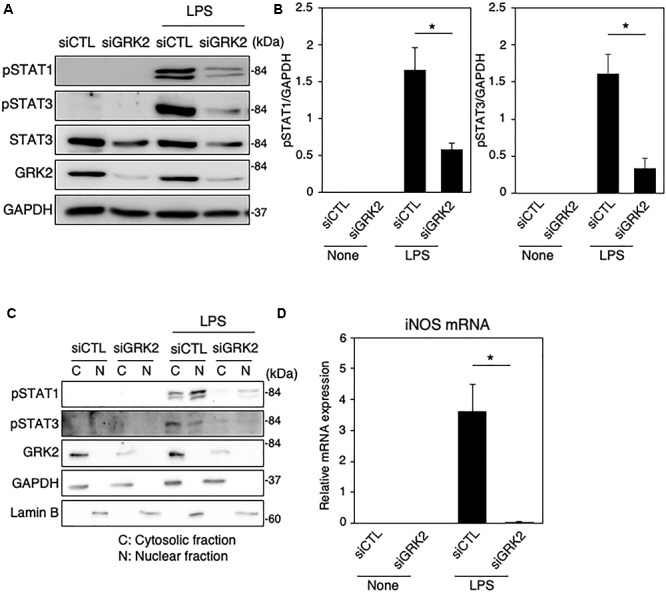
Effect of GRK2 siRNA transfection on total and phosphorylation levels of STAT1 and STAT3 in LPS-stimulated MG6 cells. **(A)** Typical Western blots of phospho-STAT1 at Tyr-701, phospho-STAT3 at Tyr-705, and total STAT3 before and 6 h after 100 ng/ml LPS application in the presence of GRK2 siRNAs (siGRK2) or the negative control siRNAs (siCTL). Transfection of siGRK2, but not of siCTL, effectively decreased GRK2 protein expression, and GAPDH was used as loading control. **(B)** Phosphorylated levels of STAT1 and STAT3 6 h after 100 ng/ml LPS application when siCTL or siGRK2 was transfected. **(C)** Cytoplasmic (C) and nuclear (N) fractions were isolated, and then phospho-STAT1 and phospho-STAT3 6 h after 100 ng/ml LPS were detected by Western blot analysis. GAPDH and lamin B served as a cytoplasmic and a nuclear marker, respectively. Shown are representative Western blots from three independent experiments in which the same results were obtained. **(D)** Effect of siGRK2 transfection on expression of iNOS mRNA 12 h after 100 ng/ml LPS application. The mRNA levels were expressed as a fold increase above control normalized GAPDH. The results represent the mean ± SEM for three independent experiments. ^∗^*P* < 0.05 by *t*-test.

To determine whether GRK2 can be involved in STAT1 and STAT3 phosphorylation in LPS-stimulated microglial cells, GRK2 siRNAs were used to knockdown microglial expression of GRK2. The ablation of GRK2 by siRNAs resulted in a significant inhibition of phosphorylation levels of STAT1 and STAT3 (Figure 3A,B). Stimulation of MG6 cells with LPS led to the translocation of phosphorylated STAT1 and STAT3 into the nucleus (Figure 3C). The nuclear translocation of phosphorylated STAT1 and STAT3 was dampened when GRK2 siRNAs were transfected. These findings suggest that GRK2 positively regulates activation of STAT1 and STAT3 in microglial cells.

As presented above, both STAT1 and STAT3 are critical regulators of iNOS expression in LPS-stimulated microglial cells. In line with our recent report (Kawakami et al., 2018), the LPS-induced increases in iNOS mRNAs were strongly prevented by transfection of GRK2 siRNAs (Figure 3D). In addition, GRK2 siRNA transfection greatly reduced the LPS-induced upregulation of mRNA levels of IL-1β, IL-6, IP-10, and IRF7 (Supplementary Figure 1).

### GRK2 Does Not Regulate Activation of NF-κB in Microglia

In MG6 cells stimulated with LPS, iNOS protein expression was strongly reduced by the NF-κB-specific inhibitor BAY 11-7082 (Supplementary Figure 2A). However, neither phosphorylation nor nuclear translocation of p65 in LPS-stimulated cells was affected when GRK2 siRNAs were transfected (Supplementary Figures 2B,C). These findings suggest that GRK2 plays no role in regulating NF-κB activation in LPS-stimulated microglial cells.

### GRK2 Regulates LPS-Stimulated Upregulation of IFN-β in Microglia

**FIGURE 4:**
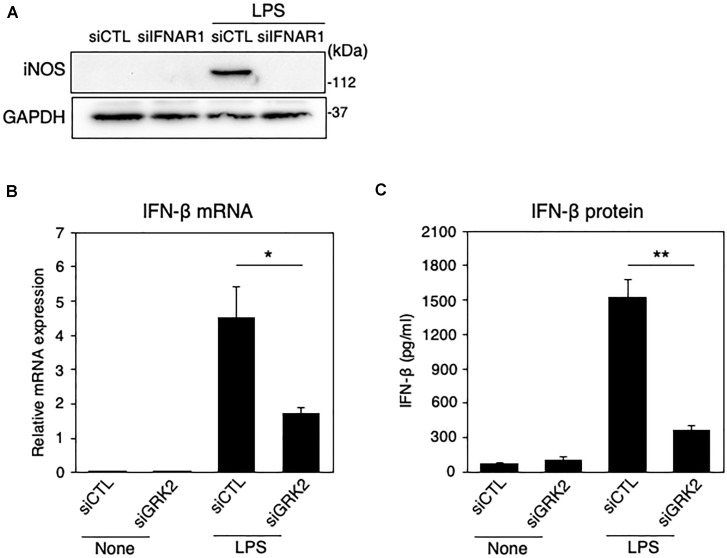
Effect of GRK2 siRNA transfection on IFN-β expression in LPS-stimulated MG6 cells. **(A)** iNOS protein expression before and 15 h after 100 ng/ml LPS application in the presence of IFNAR1 siRNAs (siIFNAR1) or the negative control siRNAs (siCTL). GAPDH served as loading control. Shown are representative Western blots from three independent experiments in which the same results were obtained. **(B)** IFN-β mRNA expression levels. Following transfection of GRK2 siRNAs (siGRK2) or the negative control siRNAs (siCTL), cells were exposed to 100 ng/ml LPS for 3 h. The mRNA levels were expressed as a fold increase above control normalized GAPDH. **(C)** Cell culture media were collected 6 h after application of 100 ng/ml LPS, and the concentrations of IFN-β were measured by the ELISA. The results represent the mean ± SEM for three independent experiments. ^∗^*P* < 0.05 and ^∗∗^*P* < 0.01 by *t*-test.

Type I IFNs, such as IFN-β, is a second major group of cytokines that are produced by LPS-activated immune cells (Noppert et al., 2007), and signal through the JAK/STAT pathway to stimulate nuclear gene expression (Horvath, 2004). Type I IFN can signal through forming a ternary complex with the type I IFN receptor, composed of its two subunits IFNAR1 and IFNAR2 (Lee and Ashkar, 2018). The ablation of IFNAR1 by siRNAs resulted in a disappearance of iNOS expression in LPS-challenged MG6 cells (Figure 4A). When LPS was applied to MG6 cells, gene expression levels of IFN-β were greatly upregulated (Figure 4B). Furthermore, when the amounts of IFN-β in culture media were measured by ELISA, LPS challenge resulted in a striking increase in IFN-β protein levels (Figure 4C). The increased mRNA and protein levels of IFN-β were significantly suppressed by transfection of GRK2 siRNAs (Figure 4A,B). These data suggest that GRK2 contributes to the production of IFN-β in LPS-stimulated microglial cells.

### GRK2 Regulates Activation of IRF1, Without Affecting TLR4 Endocytosis in LPS-Stimulated Microglia

**FIGURE 5:**
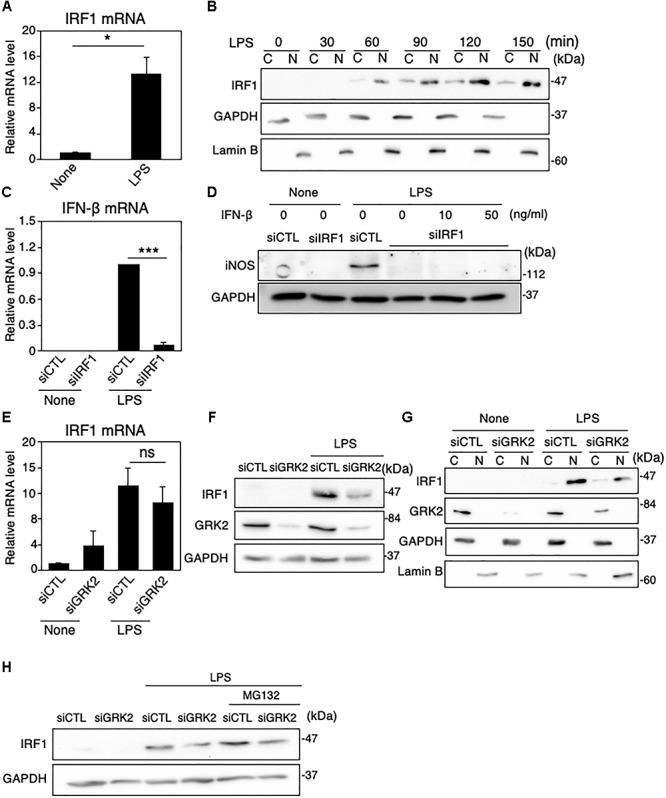
Role of IRF1 in IFN-β-mediated iNOS expression in LPS-stimulated MG6 cells. **(A)** Expression of IRF1 mRNA 3 h after 100 ng/ml LPS application. **(B)** Cytoplasmic (C) and nuclear (N) fractions were isolated, and then the time course of changes in IRF1 levels in each fraction after 100 ng/ml LPS application was tracked by Western blot analysis. **(C)** Effect of IRF1 siRNAs (siIRF1) on expression of IFN-β mRNA 3 h after 100 ng/ml LPS application. **(D)** Effect of transfection of siIRF1 on iNOS protein expression 12 h after challenge with 100 ng/ml LPS in the presence or absence of 10 or 50 ng/ml IFN-β. **(E)** Effect of GRK2 siRNAs (siGRK2) on IRF1 mRNA 3 h after 100 ng/ml LPS application. The mRNA levels were expressed as a fold increase above control normalized GAPDH. **(F)** Effect of siGRK2 on IRF1 protein 1 h after 100 ng/ml LPS. **(G)** Effect of siGRK2 on IRF1 levels in C and N fractions before and 1 h after 100 ng/ml LPS was tracked by Western blot analysis. **(H)** Influence of MG132 on the siGRK2 effect on IRF1 protein expression 90 min after 100 ng/ml LPS. MG132 at a concentration of 2 μM was added 30 min after LPS. All experiments were compared with those when the negative control siRNAs (siCTL) was transfected. GAPDH served as loading control and lamin B was used as a nuclear marker. Shown are representative Western blots from three independent experiments in which the same results were obtained. The bar graph results represent the mean ± SEM for three independent experiments. Ns, not significant. ^∗^*P* < 0.05 and ^∗∗∗^*P* < 0.001 by *t*-test.

IRFs, a family of transcription factors, play a central role in controlling the type I IFN induction at the gene transcriptional level (Honda et al., 2006). In microglial cells activated with LPS and other inflammatory stimuli, IRF1 also appears to be an essential transcription factor for expression of inflammatory mediators, including iNOS (Lee et al., 2001; Jantaratnotai et al., 2013; Matsuda et al., 2015). In MG6 cells, LPS challenge led to the upregulation of IRF1 mRNAs and the translocation of IRF1 proteins into the nucleus (Figure 5A,B). Transfection of IRF1 siRNAs strikingly eliminated LPS-induced upregulation of IFN-β mRNA expression (Figure 5C). Furthermore, IRF1 siRNA transfection negated LPS-induced iNOS expression (Figure 5D). These findings imply that IRF1 plays a crucial role in induction of IFN-β and subsequent production of iNOS in LPS-stimulated microglial cells.

A previous study with IRF3-deficient mice has established a key role for IRF3 in LPS-induced IFN-β gene expression (Sakaguchi et al., 2003). We tested whether IRF3 could be involved in induction and/or activation of IRF1 in LPS-stimulated MG6 cells. Transfection of IRF3 siRNAs did not substantially alter the LPS-induced increases in expression and nuclear translocation of IRF1 (Supplementary Figures 3A,B).

TRIF is essential for TLR-3 or TLR-4-mediated MyD88-independent pathways and contributes to pro-inflammatory cytokine production and, most importantly, induces type I IFN production, particularly IFN-β (Takeda and Akira, 2005). However, transfection of TRIF siRNAs had little effect on LPS-induced upregulation of IRF1 mRNA expression (Supplementary Figure 3C), indicating that LPS-stimulated induction of IRF1 is independent of the TRIF pathway. On the other hand, BAY 11-7082 greatly inhibited the LPS-induced increase in both mRNA and protein expression (Supplementary Figures 3D,E), which suggests that IRF1 is transcriptionally regulated by NF-κB. When GRK2 siRNAs were transfected, the LPS-induced upregulation of IRF1 mRNA was not substantially affected (Figure 5E), but that of IRF1 protein was evidently reduced (Figure 5F). In addition, the nuclear translocation of IRF1 was strongly reduced by GRK2 siRNA transfection (Figure 5G). The ability of GRK2 siRNAs to reduce the LPS-induced upregulation of IRF1 protein was evident regardless of whether the proteasome inhibitor MG132 was present, although the upregulation of IRF1 protein by LPS was markedly increased in the presence of MG132 (Figure 5H). These results suggest that GRK2 participates in LPS-induced upregulation and activation of IRF1 protein in microglial cells in a manner independent of the proteasome degradation route.

LPS-induced TLR4 endocytosis is necessary to initiate the TRIF-dependent IFN-β expression (Kagan et al., 2008). We thus examined whether GRK2 can regulate endocytosis of TLR4 in LPS-stimulated MG6 cells. FACS analysis showed much lower surface expression of TLR4 when MG6 cells were stimulated with LPS (Supplementary Figure 3F). Transfection of GRK2 siRNAs was without effect on the LPS-induced decrease in TLR4 surface expression, suggesting that GRK2 plays no regulatory role in the endocytosis pathway that delivers TLR4 to endosomes.

### GRK2 Regulates TLR9-Mediated IFN-β Expression in Microglia Through IRF1 Upregulation

**FIGURE 6:**
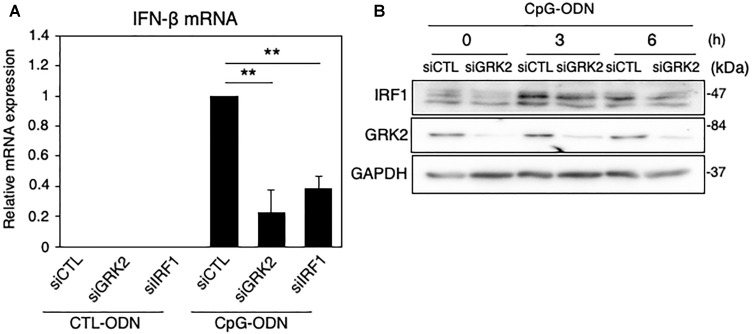
Effect of GRK2 siRNA transfection on TLR9-mediated IRF1 and IFN-β expression in MG6 cells. **(A)** IFN-β mRNA expression 3 h after 1 μM CpG-ODN or control ODN (CTL-ODN) application in the presence of GRK2 siRNAs (siGRK2), IRF1 siRNAs (siRF1) or the negative control siRNAs (siCTL). The mRNA levels were expressed as a fold increase above control normalized GAPDH and the results represent the mean ± SEM for three independent experiments. ^∗∗^*P* < 0.01 by *t*-test. **(B)** Changes in protein expression of IRF1 and GRK2 after 1 μM CpG-ODN challenge in the presence of GRK2 siRNAs (siGRK2) or the negative control siRNAs (siCTL). GAPDH served as loading control. Shown are representative Western blots from three independent experiments in which the same results were obtained.

IRF1 has been known to control TLR9-mediated IFN-β production in myeloid dendritic cells (Schmitz et al., 2007). When TLR9 was activated with CpG-ODN, a synthetic oligodeoxynucleotide containing specific unmethylated CpG motifs, the mRNA levels of IFN-β were strikingly upregulated in MG6 cells (Figure 6A). The CpG-ODN-induced increase in IFN-β mRNAs were significantly inhibited by transfection of GRK2 siRNAs or IRF1 siRNAs. Furthermore, the knockdown of GRK2 by siRNAs attenuated CpG-ODN-induced IRF1 protein expression (Figure 6B). These findings suggest that GRK2 can positively regulate TLR9-mediated IFN-β production by upregulating expression of IRF1.

### GRK2 Regulates Paclitaxel-Derived TLR4 Signaling for iNOS Expression in Microglia

**FIGURE 7:**
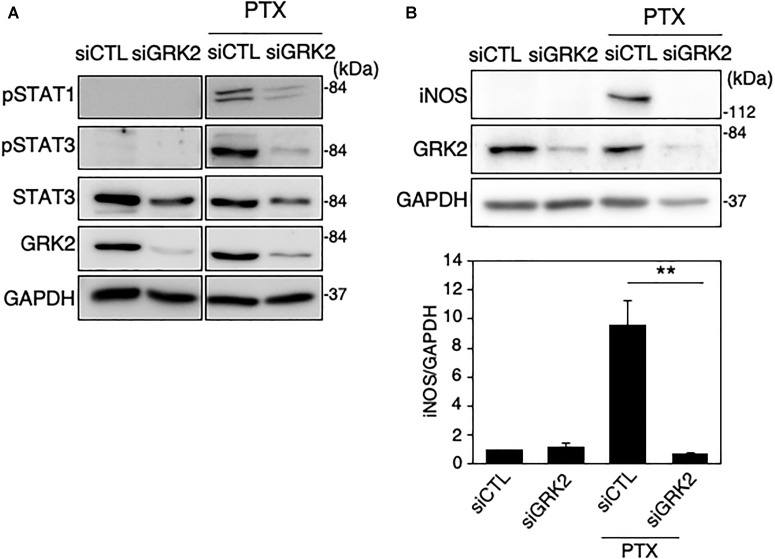
Effect of GRK2 siRNA transfection on paclitaxel-induced TLR4 signaling for iNOS expression in MG6 cells. **(A)** Phospho-STAT1 at Tyr-701, phospho-STAT3 at Tyr-705, and total STAT-3 before and 6 h after 10 μM paclitaxel (PTX) application in the presence of GRK2 siRNAs (siGRK2) or the negative control siRNAs (siCTL). Transfection of siGRK2, but not of siCTL, effectively decreased GRK2 protein expression, and GAPDH was used as loading control. Shown are representative Western blots from three independent experiments in which the same results were obtained. **(B)** Effect of siGRK2 transfection on iNOS protein expression 12 h after 5 μM paclitaxel application. The results represent the mean ± SEM for three independent experiments. ^∗∗^*P* < 0.01 by *t*-test. In the upper side, typical Western blots are shown. GAPDH served as loading control.

Paclitaxel, an anti-microtubule agent with anti-tumoral activity, is identified as a ligand to activate TLR4 signaling (Byrd-Leifer et al., 2001; Zimmer et al., 2008). We examined whether GRK2 is involved in paclitaxel-derived TLR4 signaling for iNOS expression in microglial cells. MG6 cells were challenged with paclitaxel at concentrations of 5–10 μM. Phosphorylated levels of STAT1 and STAT3 were highly upregulated as seen in LPS-stimulated cells, all of which were hampered by transfection of GRK2 siRNAs (Figure 7A). Furthermore, GRK2 siRNA transfection significantly suppressed the paclitaxel-induced increase in iNOS synthesis (Figure 7B).

### GRK2 Regulates TLR3 Signaling for iNOS Expression in Microglia

**FIGURE 8:**
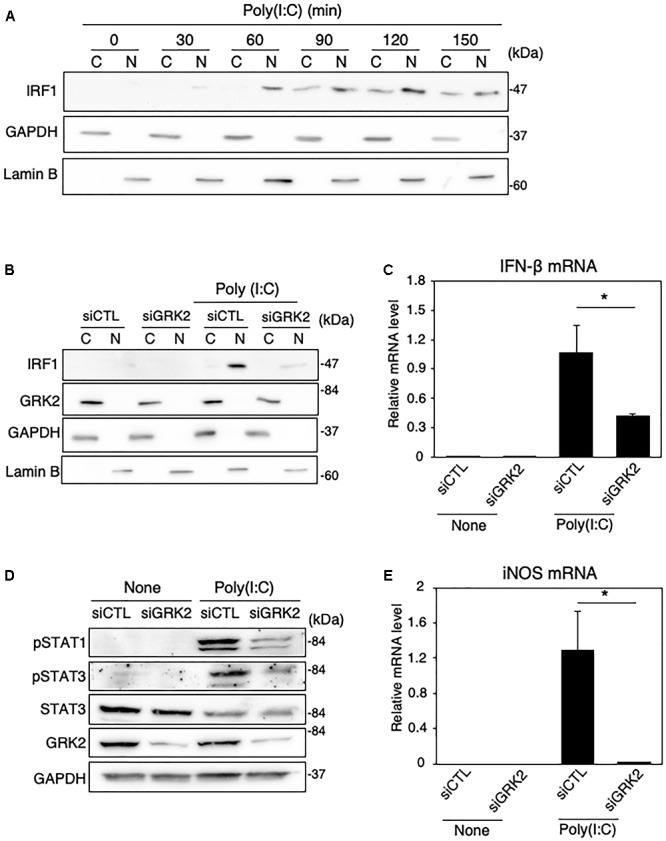
Effect of GRK2 siRNA transfection on TLR3-mediated signaling for iNOS expression in MG6 cells. **(A)** Cytoplasmic (C) and nuclear (N) fractions were isolated, and then the time course of changes in IRF1 levels in each fraction after 50 μg/ml poly(I:C) application was tracked by Western blot analysis. **(B)** Effect of transfection of GRK2 siRNAs (siGRK2) on nuclear translocation of IRF1 60 min after challenge with 50 μg/ml poly(I:C) was compared with that when the negative control siRNAs (siCTL) was transfected. GAPDH and lamin B served as a cytoplasmic and nuclear marker, respectively. **(C)** Effect of siGRK2 transfection on IFN-β mRNA expression levels 3 h after 50 μg/ml poly(I:C) application. **(D)** Effect of siGRK2 transfection on phospho-STAT1 at Tyr-701, phospho-STAT3 at Tyr-705, and total STAT-3 4 h after 50 μg/ml poly(I:C) application. GAPDH served as loading control. Shown are representative Western blots from three independent experiments in which the same results were obtained. **(E)** Effect of siGRK2 transfection on expression of iNOS mRNA 12 h after 50 μg/ml poly(I:C) application. The mRNA levels were expressed as a fold increase above control normalized GAPDH. The results represent the mean ± SEM for three independent experiments. ^∗^*P* < 0.05 by *t*-test.

Polyinosinic-polycytidylic acid [poly(I:C)] is a synthetic analog of double-stranded RNA recognized by TLR-3 (Matsumoto and Seya, 2008). We examined whether GRK2 plays a regulatory role in TLR3-mediated signaling for iNOS synthesis in microglial cells. When poly(I:C) (50 μg/ml) was applied to MG6 cells, the translocation of IRF1 into the nucleus occurred in a time-dependent manner (Figure 8A). Transfection of GRK2 siRNAs blocked the nuclear translocation of IRF1 in poly(I:C)-stimulated cells, as seen in LPS-stimulated cells (Figure 8B). In poly(I:C)-challenged cells, GRK2 siRNA transfection also significantly declined the upregulation of IFN-β mRNA levels (Figure 8C) and reduced the increases in phosphorylated levels of STAT1 and STAT3 (Figure 8D). As expected from the involvement of STAT1/3 in iNOS expression in LPS-stimulated MG6 cells, poly(I:C) administration led to the production of iNOS mRNA, which was blocked by treatment with GRK2 siRNAs (Figure 8E). In addition, GRK2 siRNA transfection greatly reduced the poly(I:C)-induced upregulation of mRNA levels of IL-1β, IL-6, IP-10, and IRF7 (Supplementary Figure 4). These results suggest that GRK2 serves as a key player for TLR3 signaling to produce iNOS in microglial cells.

### GRK2 Regulates STAT1/3 Activation in Microglia Supplemented With IFN-β

**FIGURE 9:**
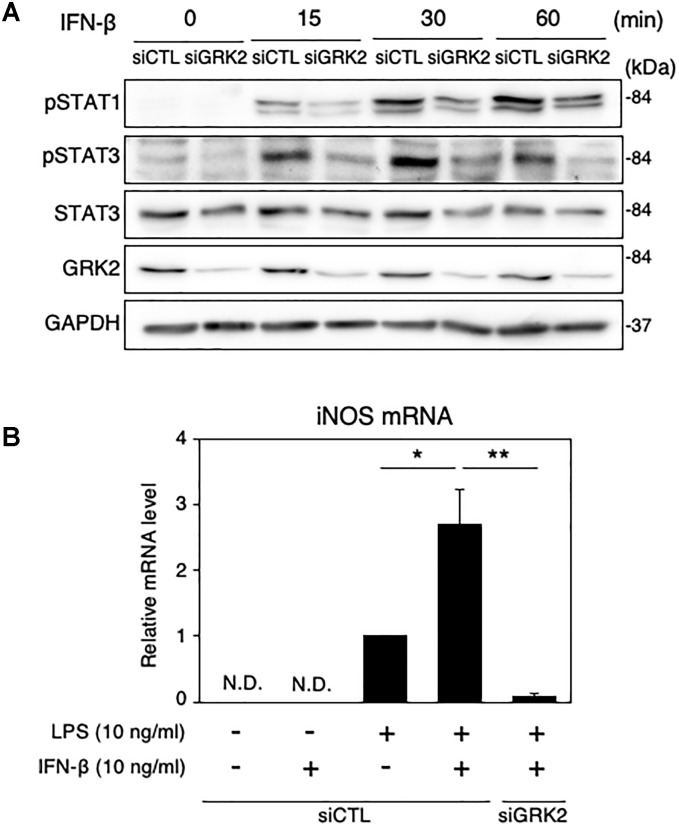
Effect of GRK2 siRNA transfection on phosphorylation levels of STAT1 and STAT3 and mRNA levels of iNOS in MG6 cells supplemented with exogenous IFN-β. **(A)** Time course of changes in phospho-STAT1 at Tyr-701, phospho-STAT3 at Tyr-705, and total STAT3 after supplementation with 10 ng/ml IFN-β. Transfection of siGRK2, but not of siCTL, effectively decreased GRK2 protein expression, and GAPDH was used as loading control. Shown are representative Western blots from three independent experiments in which the same results were obtained. **(B)** Effect of siGRK2 transfection on expression of iNOS mRNA 12 h after 10 ng/ml IFN-β supplementation in the presence of 10 ng/ml LPS. The mRNA levels were expressed as a fold increase above control normalized GAPDH. The results represent the mean ± SEM for four independent experiments. N.D., not detected. ^∗^*P* < 0.05, ^∗∗^*P* < 0.01 by *t-*test.

We also examined the role of GRK2 in STAT1/3-mediated iNOS production in microglial cells supplemented with exogenous IFN-β. IFN-β supplementation (10 ng/ml) caused time-dependent increases in phosphorylated levels of STAT1 and STAT3 in MG6 cells (Figure 9A). These changes were greatly attenuated by transfection of GRK2 siRNAs (Figure 9A). Only exposure to IFN-β failed to induce iNOS mRNAs (Figure 9B). This suggests that IFN-β by itself was not sufficient to stimulate induction of iNOS expression. However, exogenously applied IFN-β markedly augmented iNOS mRNA levels caused by a low dose of LPS (Figure 9B). GRK2 siRNA transfection blocked the iNOS mRNA expression in the presence of low-dose LPS and IFN-β (Figure 9B), providing evidence to support a role of GRK2 in IFN-β-induced activation of STAT1/STAT3 to augment iNOS production.

### GRK2 Inhibitor Reduces STAT1/3 Activation but Not IRF1 Activation in Microglia

**FIGURE 10:**
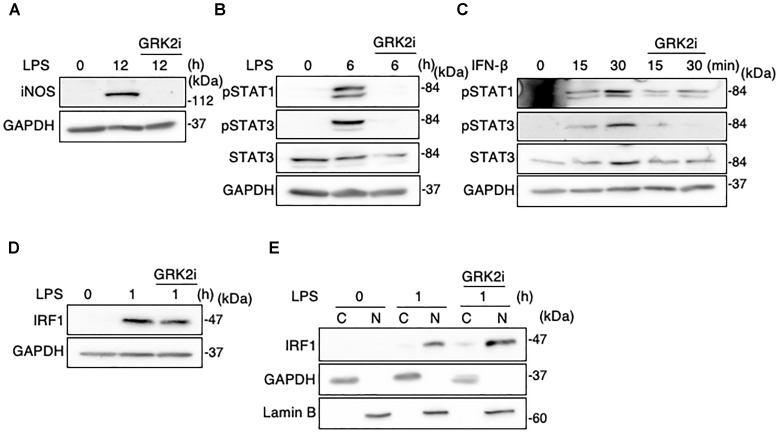
Effect of GRK2 inhibitor treatment on iNOS expression, STAT1/3 activation, and IRF1 activation in microglial cells. GRK2 inhibitor at a concentration of 10 μM was added at 30 min before challenge with 100 ng/ml LPS or 10 ng/ml IFN-β. **(A)** Expression of iNOS protein before and 15 h after LPS application. **(B)** STAT1 and STAT3 phosphorylation before and 6 h after LPS application. **(C)** STAT1 and STAT3 phosphorylation before and 15–30 min after IFN-β supplementation. **(D)** Expression of IRF1 expression before and 1 h after LPS application. **(E)** Cytoplasmic (C) and nuclear (N) fractions were isolated, and then changes in IRF1 levels in each fraction before and 1 h after LPS application was tracked by Western blot analysis. GAPDH served as loading control and lamin B was used as a nuclear marker. Shown are representative Western blots from three independent experiments in which the same results were obtained.

Methyl 5-[2-(5-nitro-2-furyl)vinyl]-2-furoate acts as a selective inhibitor of kinase activity of GRK2 (Iino et al., 2002). We examined whether this GRK2 inhibitor can modify the function or expression of STAT1/3 and IRF1 in LPS-stimulated MG6 cells. As demonstrated in our recent report (Kawakami et al., 2018), treatment with the GRK2 inhibitor abrogated the LPS-induced upregulation of iNOS protein (Figure 10A). Furthermore, GRK2 inhibitor treatment strongly eliminated phosphorylation of STAT1 and STAT3 after LPS stimulation (Figure 10B). The IFN-β-induced increases in STAT1 and STAT3 phosphorylation were also attenuated by the GRK2 inhibitor (Figure 10C). However, GRK2 inhibitor treatment did not substantially affect expression and nuclear translocation of IRF1 following LPS challenge (Figure 10D,E). These results suggest that STAT1/3, but not IRF1, in activated microglia can be regulated by GRK2 in a kinase-dependent manner.

## Discussion

**FIGURE 11:**
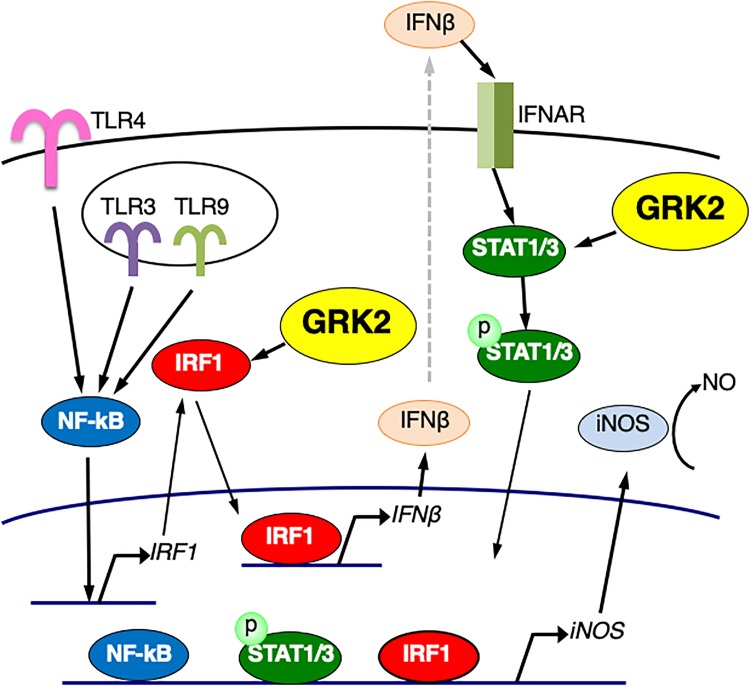
Schematic diagram of the regulatory role of GRK2 in TLRs-mediated signaling pathways for iNOS expression in microglial cells. GRK2 positively regulates not only the expression/activation of IRF1 but the activation of STAT1/3, leading to the induction of iNOS transcription. See text for details.

In this work, we delineated the TLR signaling pathway for iNOS expression in microglial cells. When TLR4 is activated by LPS, TRIF-independent signaling activates IRF-1, leading to expression of IFN-β. IFN-β, via activation of its specific receptors, employs STAT1/3 signal transduction for nuclear signaling, which in turn could enhance iNOS expression (Figure 11). In this study, we found that LPS challenge led to the induction of IRF1 expression and the translocation of IRF1 into the nucleus in microglial cells. Stimulation of TLR9 with CpG-ODN also increased IRF1 expression and led to upregulate IFN-β transcription. Furthermore, LPS-induced upregulation of IFN-β and iNOS mRNA expression was eliminated by transfection of IRF1 siRNAs, suggesting that, in LPS-stimulated microglia, IRF1 is one of essential transcription factors for expression of IFN-β to augment iNOS production. In accordance with this finding, IRF1 has been implicated in the induction of inflammatory mediators, including iNOS, in microglial cells activated with LPS and other inflammatory stimuli (Lee et al., 2001; Jantaratnotai et al., 2013; Matsuda et al., 2015).

GRK2 belongs to the GRK family to regulate the activity of GPCRs, of which GRK2 is ubiquitously expressed and play multiple roles in cell signaling beyond GPCR desensitization (Penela et al., 2010, 2014; Evron et al., 2012; Mayor et al., 2018; Nogues et al., 2018). Thus, GRK2 is not only capable of interacting with a variety of endocytic proteins, but it can modulate various signaling cascades in a kinase-independent fashion to affect a wide range of cellular processes (Evron et al., 2012; Mayor et al., 2018; Nogues et al., 2018). Our recent study has shown that GRK2 controls reactive oxygen species production pathway in LPS-stimulated microglial cells (Kawakami et al., 2018). In this study, our results with the use of GRK2 siRNAs demonstrate that the protein expression and nuclear translocation of IRF1 is positively regulated by GRK2 when TLR4 is activated by LPS in microglial cells. It appeared likely that GRK2 is involved in LPS-induced upregulation and activation of IRF1 protein in microglial cells without affecting the proteasome degradation route. TLR4 activates MyD88-dependent signaling mainly at plasma membranes and TRIF-dependent signaling at the endosomal membrane after internalization of TLR4 complex into endosomes whereupon TRIF is recruited (Rajaiah et al., 2015). We showed that GRK2 did not participate in the initiation of events that promote TLR4 endocytosis. These new findings suggest a novel role of GRK2 in TLR4-induced inflammatory signaling in microglial cells and provide promising new insight into the molecular control of GRK2.

TLR3 triggers activation of the MyD88-independent and TRIF-dependent signaling pathway leading to IFN-β production (Yamamoto et al., 2003; Kawai and Akira, 2010; Kawasaki and Kawai, 2014). We found that activation of TLR3 with poly(I:C) promoted the translocation of IRF1 into the nucleus and upregulated transcription of IFN-β in microglial cells. Transfection of GRK2 siRNAs blocked the poly(I:C)-induced nuclear translocation of IRF1, leading to the reduced upregulation of IFN-β expression. We interpret these observations to indicate that the TLR3-mediated induction of IFN-β transcription is highly regulated by GRK2 through its ability to activate IRF1 (Figure 11). Alternatively, it seems unlikely that the ability of GRK2 to interfere with MyD88 or TRIF, if any, contributes to its regulatory role in TLRs-mediated iNOS induction.

While LPS activation of TLR4 in immune cells induces expression of multiple pro-inflammatory cytokines, including tumor necrosis factor-α and IL-1β, through the MyD88-dependent signaling pathway, type I IFNs, such as IFN-β, is a second major group of cytokines that are produced by the MyD88-independent and TRIF-dependent signaling pathway in LPS-activated immune cells (Akira and Takeda, 2004; Noppert et al., 2007). We demonstrated that both STAT1 and STAT3 were greatly activated in LPS-stimulated microglial cells, although STAT3 and STAT1 appear to be constitutive and inducible, respectively. The same results were found when microglia were challenged with poly(I:C). In light of the notion that type I IFNs signal through the JAK/STAT pathway to stimulate nuclear gene expression (Horvath, 2004; Ivashkiv and Donlin, 2014), it would be reasonable to consider that IRF1, which is induced by TRIF-independent fashion, promotes the production of IFN-β downstream of TLR4 signaling as well as TLR3 signaling in microglia, resulting in STAT1/3 activation and thereby iNOS production (Figure 11).

We found that transfection of GRK2 siRNAs suppressed the activation of STAT1 and STAT3 in microglial cells when TLR3 and TLR4 were stimulated with poly(I:C) and LPS or paclitaxel, respectively. This cannot be solely the result of the blockade of the IRF1 activation that is positively regulated by GRK2. It should be noted that GRK2 siRNA transfection blocked the exogenous IFN-β supplementation-induced increases in phosphorylated STAT1 levels as well as phosphorylated STAT3 levels. Therefore, we suggest that, in addition to its ability to activate IRF1, GRK2 promotes the activation of the STAT pathway, through which IFN-β signals to stimulate iNOS expression, in activated microglia (Figure 11).

The blocking effect of GRK2 siRNA transfection on STAT1 and STAT3 phosphorylation was mimicked by the GRK2 inhibitor which acts on kinase activity of GRK2, suggesting that STAT1 and STAT3 in activated microglia can be regulated by GRK2 in a kinase-dependent manner. However, GRK2 inhibitor treatment exhibited no substantial effect on expression and nuclear translocation of IRF1 following LPS challenge. Whether a direct interaction exists between GRK2 and IRF1 remains the subject of ongoing studies.

## Conclusion

In conclusion, we present evidence supporting a direct role of GRK2 in regulating TLR3-, TLR4-, and TLR-9-mediated inflammatory signaling in microglial cells. We thus show that GRK2 highly regulates the expression/activation of IRF1 as well as the activation of the STAT pathway, leading to augmented transcription of iNOS. However, the mode of action profiling of GRK2 underlying its interactions with these inflammatory signaling molecules awaits further investigation. Microglial cells are the resident tissue macrophages located in the central nervous system (CNS) and have a role in monitoring the brain for immune insults and invading pathogens (Ohashi et al., 2015). They are the major source of iNOS in the CNS (Saha and Pahan, 2006; Xanthos and Sandkuhler, 2014; Chitnis and Weiner, 2017). The upregulation of iNOS and subsequent excessive NO production are considered to play a contributory role in the pathogenesis of different neuroinflammatory diseases (Smith and Lassmann, 2002; Pannu and Singh, 2006; Ghasemi and Fatemi, 2014). Our findings highlight a novel pathophysiological role of GRK2 in regulating inflammatory signaling in microglia with a potential therapeutic window for CNS disorders in which neuroinflammation plays a critical role.

## Author Contributions

WO, TA, and YH conceived and designed the experiments. SP, MK, TS, and HY performed the experiments. SP and WO analyzed the data. SP, WO, KH, and YH wrote the article. All authors read and approved the final manuscript.

## Conflict of Interest Statement

The authors declare that the research was conducted in the absence of any commercial or financial relationships that could be construed as a potential conflict of interest.
