# Supplementary material for: Regulatory Role of GRK2 in the TLR Signaling-Mediated iNOS Induction Pathway in Microglial Cells
Source: Front Pharmacol. 2019 Feb 4;10:59. doi: 10.3389/fphar.2019.00059 (PMC6369205; doi:10.3389/fphar.2019.00059)
Supplement: Supplementary file 1 [file Data_Sheet_1.pdf]

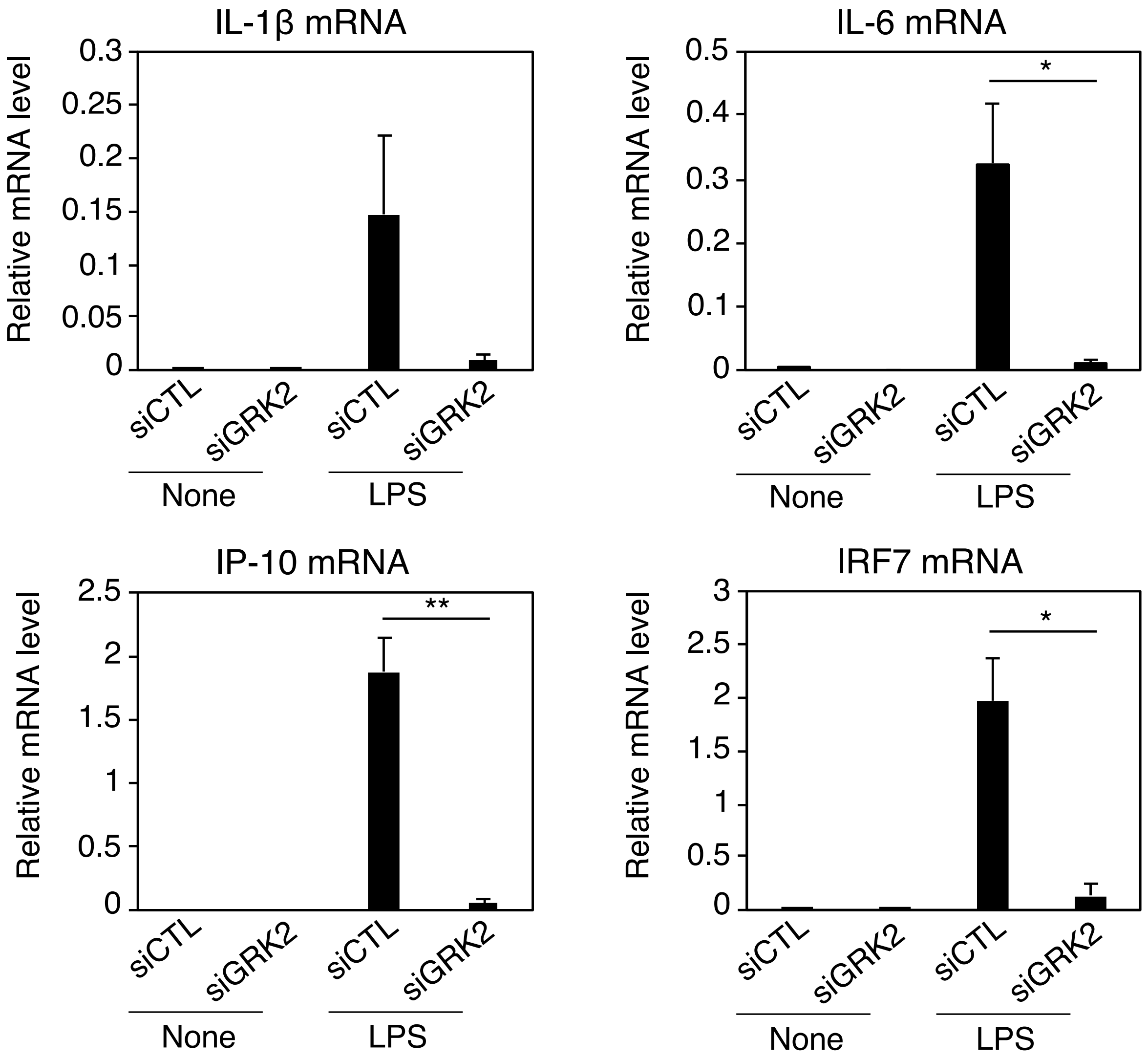

Supplementary Figure 1. Effect of GRK2 siRNA transfection on mRNA levels of IL-1β, IL-6, IP-10, and IRF7 in LPS-stimulated cells. mRNA levels 12 h after 100 ng/ml LPS. The mRNA levels were expressed as a fold increase above control normalized GAPDH. The results represent the mean ± SEM for three independent experiments. $^{*}P<0.05$ and $^{**}P<0.01$ by *t* test.

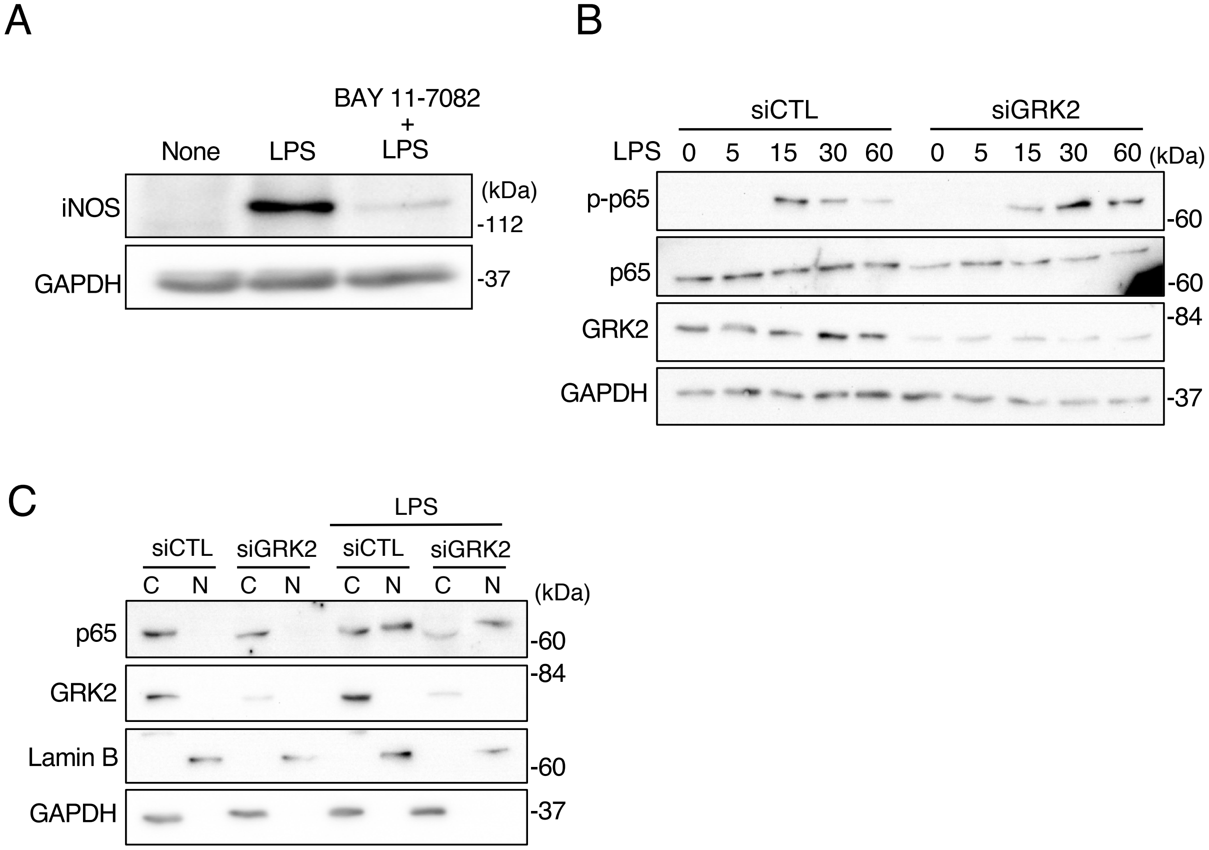

Supplementary Figure 2. Effect of GRK2 siRNA transfection on NF-κB activation in LPS-stimulated MG6 cells. (A) Effect of BAY 11-7082 on iNOS protein expression 12 h after 100 ng/ml LPS application. BAY 11-7082 at a concentration of 5 μM was added 30 min before LPS. **(B)** Time course of changes in p65 phosphorylated levels after LPS application. **(C)** Cytoplasmic (C) and nuclear (N) fractions were isolated, and then changes in p65 levels in each fraction before and 1 h after LPS in the presence of GRK2 siRNAs (siGRK2) or the negative control siRNAs (siCTL) was tracked by Western blot analysis. GAPDH served as loading control and lamin B was used as a nuclear marker.

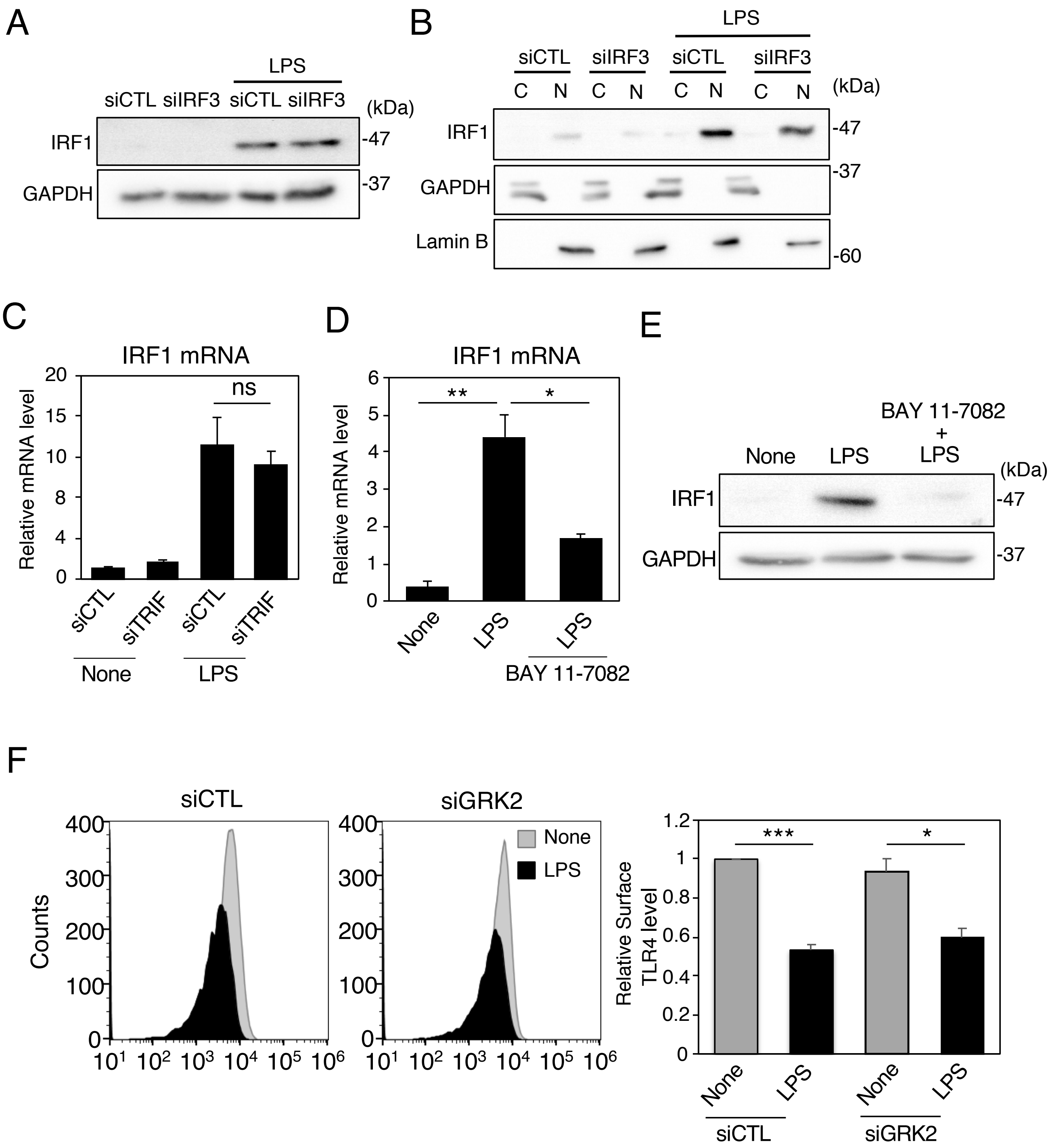

Supplementary Figure 3. IRF1 expression and activation in in LPS-stimulated MG6 cells. (A) Effect of IRF3 siRNA (siIRF3) on protein expression of IRF1 1 h after 100 ng/ml LPS. (**B**) Cytoplasmic (C) and nuclear (N) fractions were isolated, and then changes in IRF1 levels in each fraction before and 1 h after LPS in the presence of siIRF3 was tracked by Western blot analysis. **(C)** Effect of TRIF siRNAs (siTRIF) on IRF1 mRNA 3 h after LPS. (**D**) Effect of BAY 11-7082 on IRF1 mRNA levels 3 h after LPS. The mRNA levels were expressed as a fold increase above control normalized GAPDH. (**E**) Effect of BAY 11-7082 on IRF1 protein 1 h after LPS. BAY 11-7082 at 5

μM was added 30 min before LPS. **(F)** Flow cytometric analysis of surface expression of TLR4 at 3 h after LPS when siGRK2 was transfected. Left side shows representative surface TLR4 staining of MG6 cells. All experiments were compared with those when the negative control siRNAs (siCTL) was transfected. The bar graph results represent the mean ± SEM for three independent experiments. ns = not significant. $^{*}P<0.05$ and $^{***}P<0.001$ by *t* test. GAPDH served as loading control and lamin B was used as a nuclear marker.

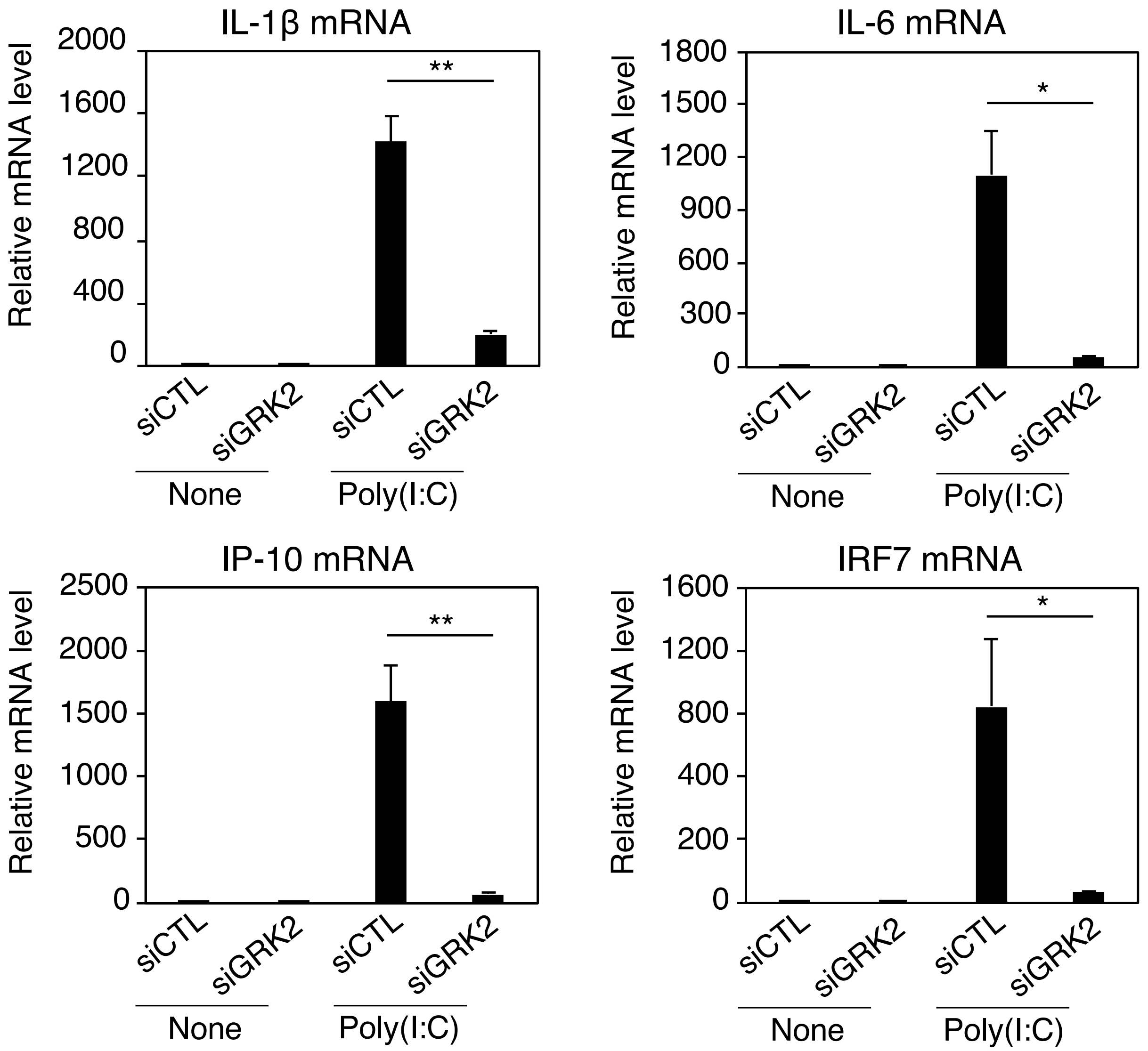

Supplementary Figure 4 Effect of GRK2 siRNA transfection on mRNA levels of IL-1β, IL-6, IP-10, and IRF7 in Poly(I:C)-stimulated cells. mRNA levels 3 h after 50 µg/ml poly(I:C). The mRNA levels were expressed as a fold increase above control normalized GAPDH. The results represent the mean ± SEM for three independent experiments. $^{*}P<0.05$ and $^{**}P<0.01$ by *t* test.
